# Supplementary material for: Chlorogenic acid inhibits NLRP3 inflammasome activation through Nrf2 activation in diabetic nephropathy
Source: PLoS One. 2025 Jan 6;20(1):e0316615. doi: 10.1371/journal.pone.0316615 (PMC11703029; doi:10.1371/journal.pone.0316615)
Supplement: S1 File — (PDF) [file pone.0316615.s002.pdf]

Fig 3. A

NLRP3

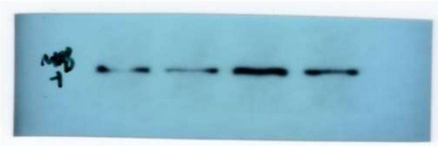

120KDa

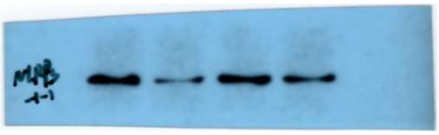

120KDa

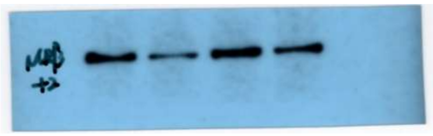

120KDa

c-capase-1

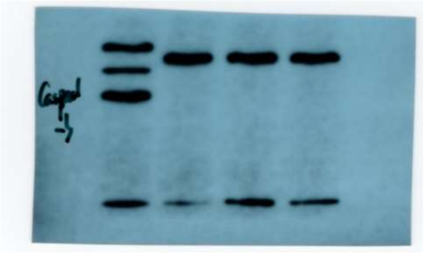

50KDa
40 KDa
30KDa
20KDa

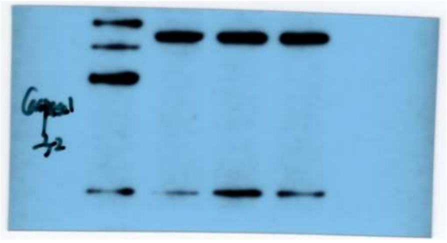

50KDa
40 KDa
30KDa
20KDa

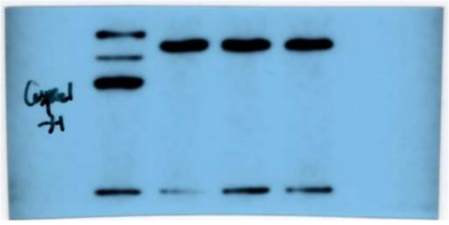

50KDa
40 KDa
30KDa
20KDa

IL-1β

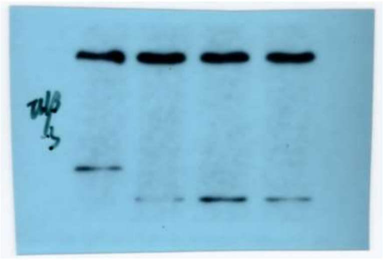

30KDa

20KDa

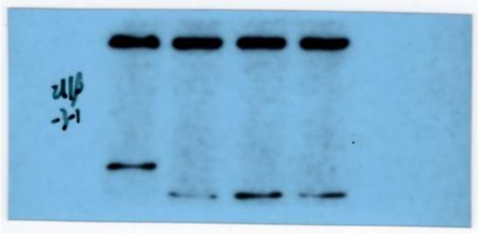

30KDa

20KDa

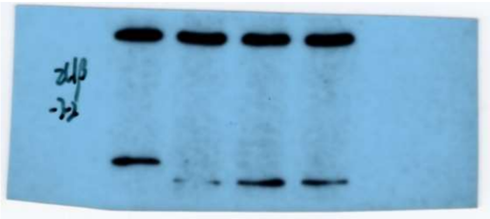

30KDa

20KDa

IL-18

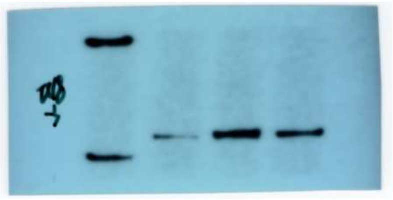

30KDa

20KDa

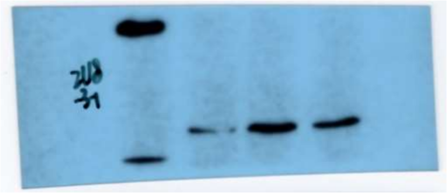

30KDa

20KDa

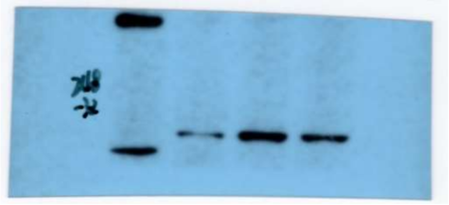

30KDa

20KDa

Fig 3. C

Nrf2

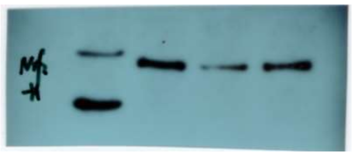

120KDa

80KDa

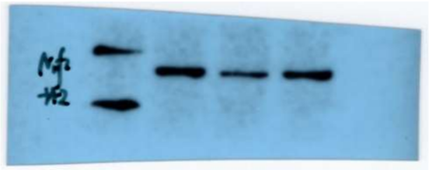

120KDa

80KDa

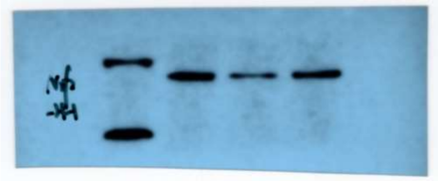

120KDa

80KDa

HO-1

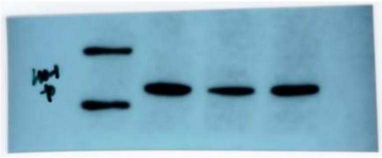

40KDa

30KDa

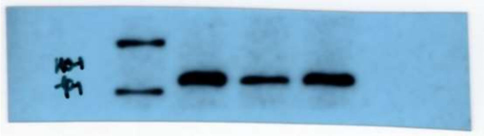

40KDa

30KDa

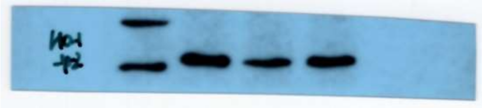

40KDa

30KDa

Fig 4. A

NLRP3

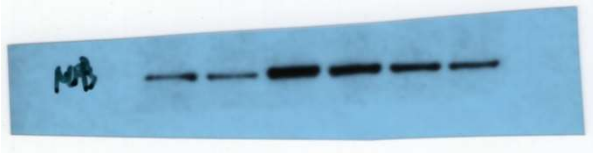

120KDa

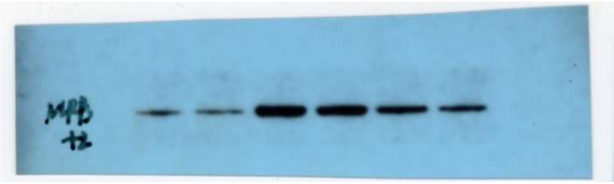

120KDa

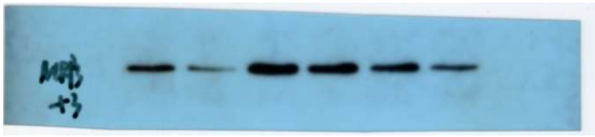

120KDa

c-capase-1

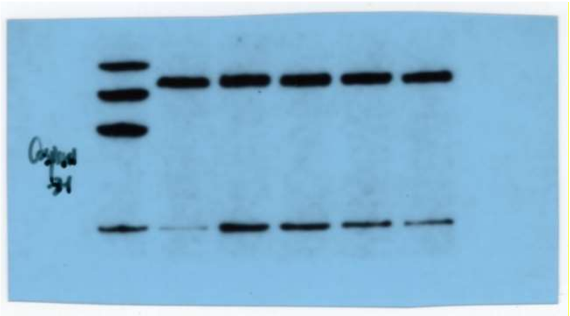

50KDa
40 KDa
30KDa
20KDa

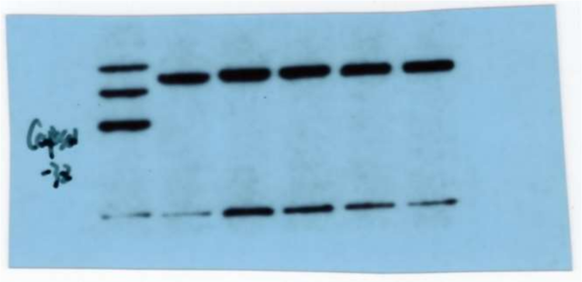

50KDa
40 KDa
30KDa
20KDa

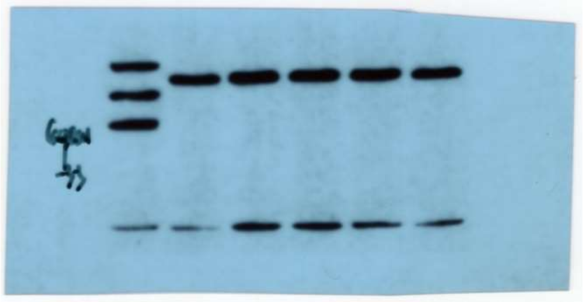

50KDa
40 KDa
30KDa
20KDa

IL-1β

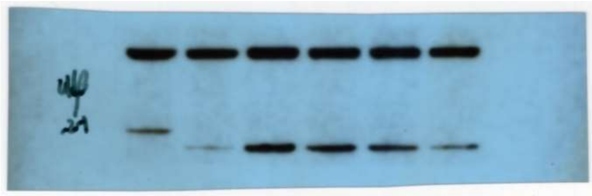

30KDa
20KDa

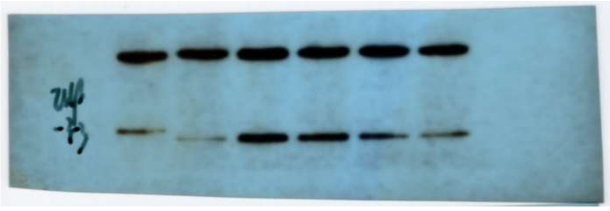

30KDa
20KDa

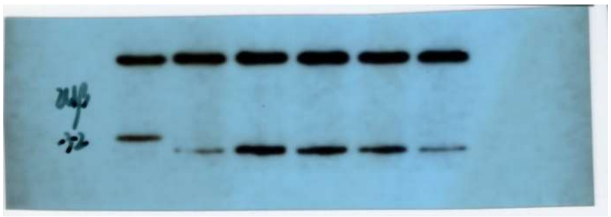

30KDa
20KDa

IL-18

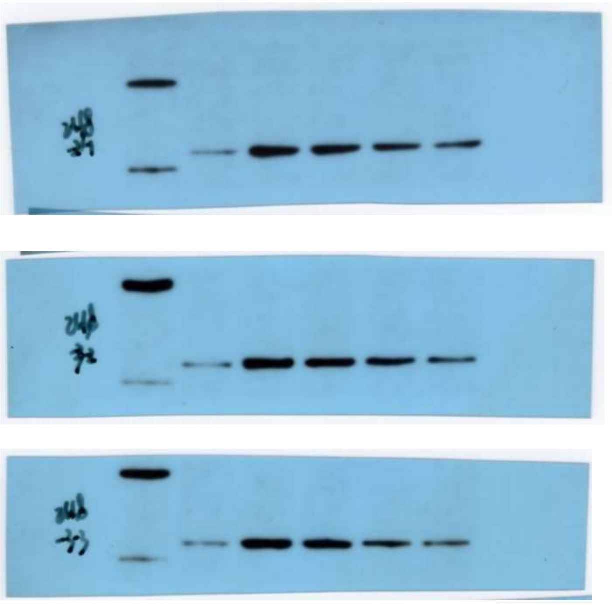

30KDa
20KDa
30KDa
20KDa
30KDa
20KDa

Fig 4. B

Nrf2

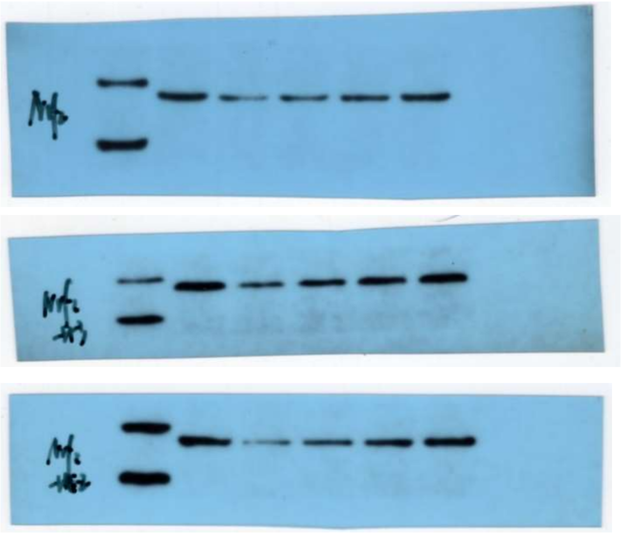

120KDa
80KDa
120KDa
80KDa
120KDa
80KDa

HO-1

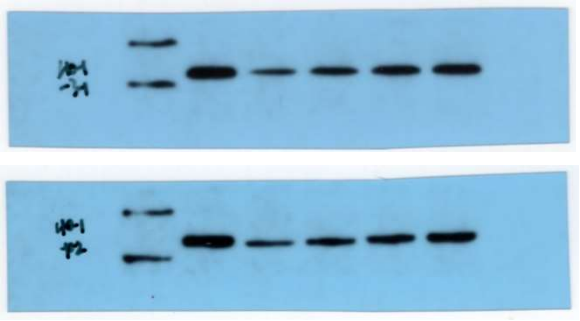

40KDa
30KDa
40KDa
30KDa

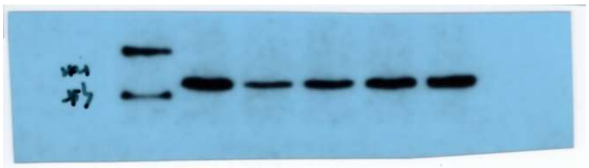

40KDa
30KDa

Fig 5

HO-1

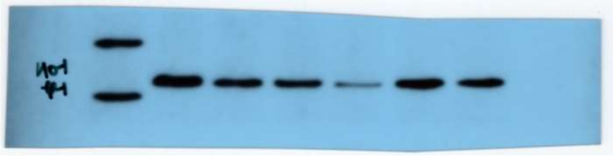

40KDa
30KDa

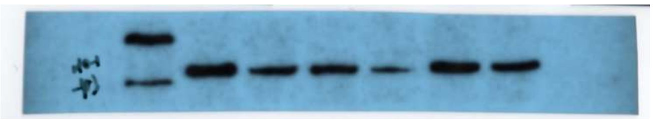

40KDa
30KDa

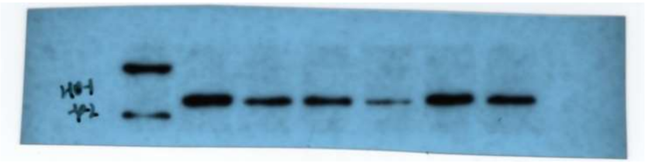

40KDa
30KDa

NLRP3

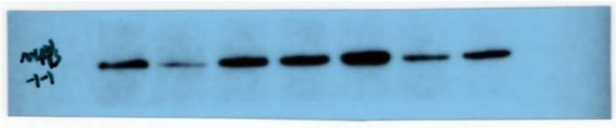

120KDa

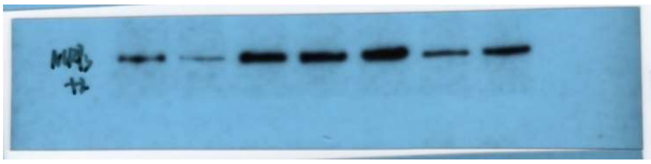

120KDa

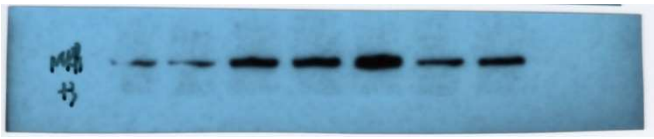

120KDa

c-capase-1

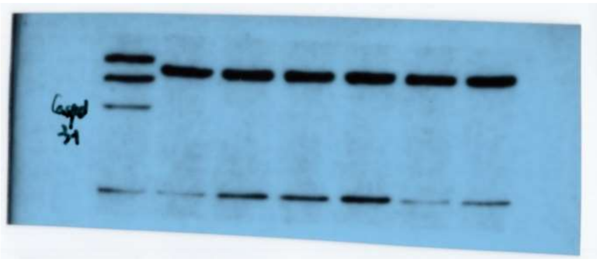

50KDa
40 KDa
30KDa
20KDa

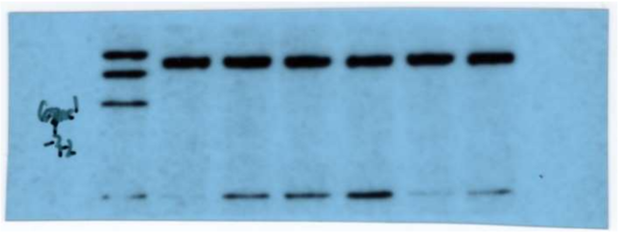

50KDa
40 KDa
30KDa
20KDa

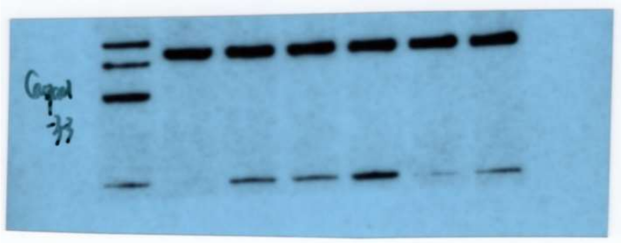

50KDa
40 KDa
30KDa
20KDa

# IL-1β

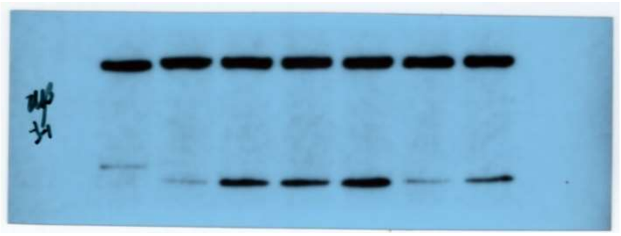

30KDa
20KDa

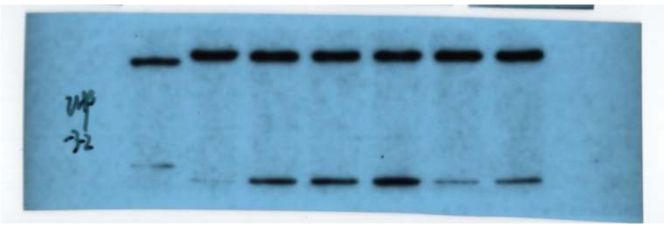

30KDa
20KDa

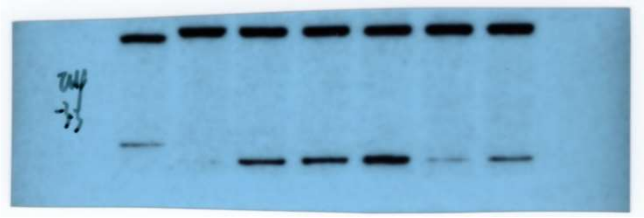

30KDa
20KDa

# IL-18

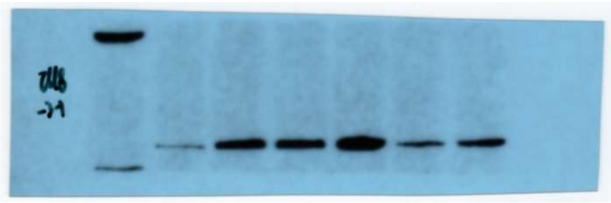

30KDa
20KDa

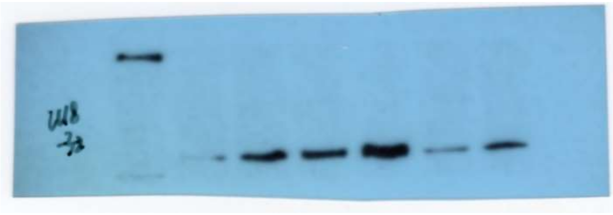

30KDa
20KDa

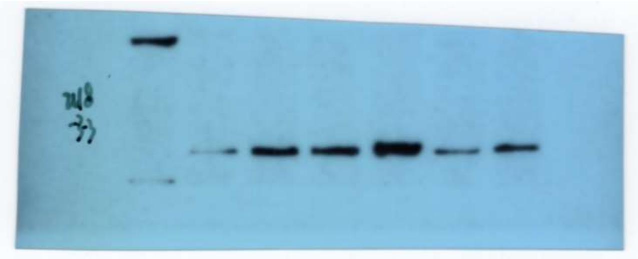

30KDa

20KDa
