# Supplementary material for: Chlorogenic acid inhibits NLRP3 inflammasome activation through Nrf2 activation in diabetic nephropathy
Source: PLoS One. 2025 Jan 6;20(1):e0316615. doi: 10.1371/journal.pone.0316615 (PMC11703029; doi:10.1371/journal.pone.0316615)
Supplement: Attachment — Submitted filename: Notes on Review for PLOS One.docx [file pone.0316615.s003.docx]

Notes on Review for PLOS One

“Chlorogenic acid inhibits NLRP3 inflammasome activation through Nrf2 activation in diabetic nephropathy”

Accept with Minor Revisions

**Review Comments to Author**:

This manuscript is technically sound, and the data supports all conclusions presented in the final document. Statistical Analysis has been performed and indicates that the results presented are statistically significant. The authors have also made it clear that all data underlying the findings in the manuscript are fully available.

**Summary**:

Diabetic neuropathy is a serious complication of both type 1 and type 2 diabetes, and it often affects the kidneys’ ability to remove waste products and extra fluid from the body. Inflammation caused by the NLRP3 inflammasome and further Nrf2 activation is a large contributing factor to the progression of diabetic nephropathy (DN). Chlorogenic acid (CGA) could activate Nrf2, which is found in nature. This study evaluated the ability of CGA to reduce inflammation. Findings showed that serum creatinine (Scr), blood urea nitrogen (BUN), and urinary protein excretions decreased after applying CGA. This occurs because CGA can activate the function of Nrf2 and halt the activity of NLRP3. Thus, this study demonstrated that chlorogenic acid could decrease DN progression and showed further that this impact is largely due to suppression of the NLRP3 inflammation and is modulated through the Nrf2 pathway. This research represents potential therapeutic implications for DN.

**Abstract**:

*Comments:* Overall, the abstract explains the background and premise of the article concisely and fully.

*Major Revisions*:

*Minor Revisions:*

1. In the abstract, you wrote: “CGA administration can active the Nrf2 pathway and inhibited NLRP3 inflammasome activation.” However, it should be noted that “CGA administration can **activate** the Nrf2 pathway and **inhibit** NLRP3 inflammasome activation.”

**Introduction**:

*Comments:*

The introduction concisely explains DN and how NLRP3 plays a role in developing the disease. It provides an adequate basis for the overall study.

*Major Revisions:*

1. The hypothesis should be rephrased; the way it is currently explained it does not explain how the Nrf2 negatively regulates NLRP3 inflammasome, particularly in this segment “negatively regulated the activation of NLRP3 inflammasome via regulation of the NLRP3 inflammasome.”

*Minor Revisions:*

None

**Methods**:

The methods described here were sufficient

*Major Revisions*: None

*Minor Revisions*:

I see that there are statistics performed on each of these experiments, but I could not find where there was a mention of how many times these experiments were completed. If they were done in duplicate or triplicate, please state this in the methods, particularly for the western blot analysis results and subsequent relative protein quantification.

**Results**:

Major Revisions: None

Minor Revisions: None

**Discussion**:

Major Revisions: None

Minor Revisions:

1. The discussion mentions that “Growing evidence has demonstrated that some natural plant compounds or herbal products have therapeutic potential via regulation of inflammation to prevent and potentially treat DN[20-22]” but does not provide further details about where these compounds are found. This section would be improved in examples of where these compounds are found were also included.
2. Some small grammar repairs, consider changing “Chlorogenic acid (CGA) with a good Nrf2 activation effect possessed anti-inflammatory property. Little information concerning CGA is available on DN, and the underlying mechanism still remain to be further revealed.”

To “Chlorogenic acid (CGA) with a good Nrf2 activation effect possessed anti-inflammatory **properties**. Little information concerning CGA is available on DN, and the underlying mechanism still **remains** to be further revealed.”
